# The Role of Lower Limb Kinetics in Boxing Punches and the Impact of Fatigue on Biomechanical Performance

**DOI:** 10.3390/bioengineering12121355

**Published:** 2025-12-12

**Authors:** Charles Stewart, Ross Cornett, Julien S. Baker, Yaodong Gu, Frédéric Dutheil, Ukadike Chris Ugbolue

**Affiliations:** 1 Faculty of Sports Science, Ningbo University, Ningbo 315211, China; b00191475@studentmail.uws.ac.uk (C.S.); juliensbakerw1@gmail.com (J.S.B.);; 2 School of Health and Life Sciences, Sport and Physical Activity Research Institute (SPARI), University of the West of Scotland, South Lanarkshire, Scotland G72 0LH, UK; 3 Centre for Health and Exercise Science Research, Department of Sport, Physical Education and Health, Hong Kong Baptist University, Kowloon Tong, Hong Kong; 4 National Centre for Scientific Research (CNRS), LaPSCo, Physiological and Psychosocial Stress, University Hospital of Clermont-Ferrand, CHU Clermont-Ferrand, Preventive and Occupational Medicine, WittyFit, Université Clermont Auvergne, 63000 Clermont-Ferrand, France

**Keywords:** boxing, ground reaction force, rate of force development, fatigue, biomechanics, kinetic chain

## Abstract

**Purpose:** This study investigated the contribution of lower limb kinetics to punch performance in amateur boxing and examined the effects of fatigue on biomechanical efficiency. **Methods:** Ten male amateur boxers performed six punch types (jab, cross, left hook, right hook, left uppercut, right uppercut) under non-fatigued and post-fatigue conditions. Ground reaction force (GRF) and rate of force development (RFD) were measured using dual force plates, while punch outputs were assessed via a boxing force sensor. Fatigue was induced using a 9.5 min lower-body circuit. **Results:** Pre-fatigue, the cross punch generated the highest outputs for punch force (1475.42 N), GRF (947.54 N), and RFD (3973.38 N/s). Post-fatigue, punch force declined significantly across all punches (–4.26%, *p* = 0.027), with the greatest reductions in the cross and left hook. RFD responses were variable, with compensatory increases observed in some punches. Intra-individual analysis revealed greater fatigue-induced declines in the weakest punches (–9.84%, *p* = 0.001) compared with the strongest (–4.63%, *p* = 0.027). **Conclusions:** Lower limb force generation, particularly rear-leg drive, is critical to punch effectiveness and fatigue resilience. Conditioning programs should prioritise lower limb endurance while addressing performance variability across punch types.

## 1. Introduction

Despite longstanding reports of physical trauma and its acute and chronic neurological and neuropsychological effects on the brain, boxing remains popular [1,2]. Boxing is a multidimensional combat sport that requires the integration of technical skill, strength, coordination and endurance to deliver high-impact strikes while maintaining defensive capability [3,4]. Such investigations across different experience levels are important, as Zazryn and colleagues [5] reported that injuries occur more frequently in amateur boxing than in professional boxing. Central to punching performance is the ability to generate force rapidly and transfer it efficiently through the kinetic chain-from the lower limbs to the upper extremities-culminating at the punch point. Ground reaction force (GRF) and rate of force development (RFD) are critical components of this process, particularly in rotational and straight-line strikes where force production originates from the lower body and trunk [6,7].

Research suggests that boxers who optimise the sequential activation of proximal-to-distal segments achieve greater punch impact [8]. However, the biomechanical literature remains disproportionately focused on upper-body kinematics, despite evidence that lower limb force contributes significantly to punching force and stability [7,9]. GRF generated by the rear leg, in particular, is a key determinant of punch power during the cross and hook [9]. RFD further reflects the explosive capacity of the legs, and its relationship with punching efficiency has been highlighted in multiple striking sports [3,10].

Fatigue is another crucial factor that may disrupt this biomechanical sequence. Lower body fatigue has been shown to reduce postural control, impair kinetic transfer and alter strike mechanics [6]. Yet, the degree to which punch performance deteriorates under fatigue-particularly in amateur boxers-remains underexplored. Some evidence indicates that boxers may maintain output through compensatory upper body adjustments [9], while others suggest significant reductions in both GRF and punch force after fatigue [7].

Few studies have examined fatigue-induced biomechanical changes across multiple punch types. The extent to which different strikes (e.g., jab vs. uppercut) are affected by fatigue may reflect the varying kinetic demands of each punch. Additionally, no previous research has compared intra-individual variability in punch performance-specifically, how a boxer’s weakest and strongest punches respond to fatigue. Understanding this variability could offer practical insight for conditioning interventions aimed at improving consistency and reducing performance drop-off.

This study aimed to examine the role of lower limb kinetics (GRF and RFD) in six common boxing punches-jab, cross, left hook, right hook, left uppercut and right uppercut-and to determine the effect of lower body fatigue on punch output. A novel element of this investigation was the intra-subject comparison between each boxer’s strongest and weakest punches. Findings from this study are intended to inform training strategies that enhance lower limb endurance and minimise intra-individual variability in strike performance.

## 2. Materials and Methods

### 2.1. Study Design

**Figure 1:**
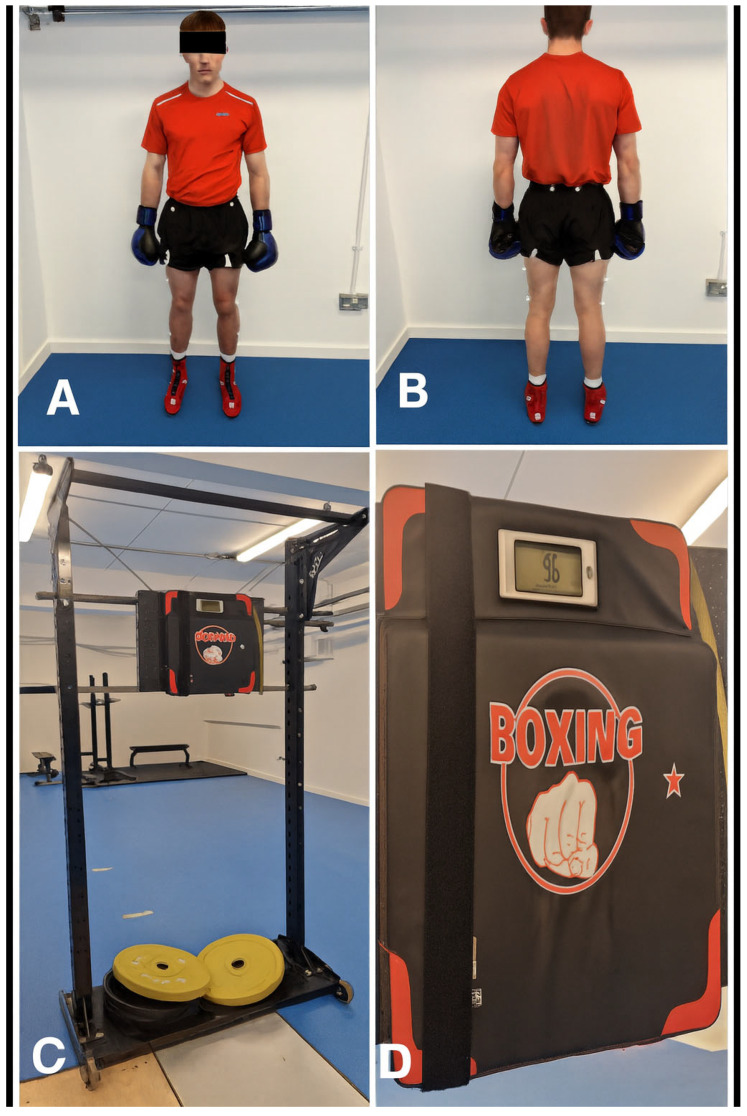
Experimental setup for punch force assessment. (**A**) Front view of a participant in the standardized stance position wearing boxing gloves and displaying the lower limb Plug-in Gait (PiG) retro-reflective marker set., (**B**) Back view of participant stance with boxing glove with (PiG) marker set, (**C**) Display of the mounted boxing pad attached to a fixed frame for stability, and (**D**) Digital display used to record punch force values.

A within-subject repeated-measures design was employed to investigate the contribution of lower limb kinetics in punching performance and the effect of fatigue on this relationship. Each participant completed two testing conditions: a non-fatigued punch assessment and a post-fatigue punch assessment conducted immediately after a standardised lower limb fatigue circuit. This design controlled for inter-individual variability and enabled direct comparison of biomechanical outputs under fatigued and non-fatigued states. All testing was conducted under standardised conditions in a university biomechanics laboratory (Figure 1).

### 2.2. Participants

Ten right-hand-dominant male amateur boxers (mean age: 25.2 ± 3.8 years; height: 178.6 ± 6.5 cm; body mass: 78.4 ± 7.9 kg) were recruited from local boxing gyms and university sport science networks. All participants had a minimum of two years’ boxing experience and were free from injury at the time of data collection. Ethical approval was obtained from the University of the West of Scotland Ethics Committee (Approval Number: 15871) and written informed consent was secured from all participants.

### 2.3. Kinetic Data Collection

Kinetic data were collected using a Kistler force plate system (Kistler Instruments Ltd., Hampshire, UK), sampling at 1000 Hz. Participants adopted their natural boxing stance, with each foot positioned on a force plate throughout the punch execution. Ground reaction force (GRF) data were recorded across three axes: vertical (Fz), anterior-posterior (Fy) and medial-lateral (Fx). Primary analyses focused on the Fz and Fy components due to their relevance to propulsion and force transmission [11].

Rate of force development (RFD) was calculated using the standard formula:

RFD = (ΔForce (N))/(ΔTime (s))

This provided a measure of the explosive capacity of force production during punching actions. Kinetic output was segmented into four distinct phases: Base, Load, Drive and Impact. All testing was performed at the same time of day to control for the effects of diurnal variation.

### 2.4. Punch Force Output

The examination of force distribution and centre of pressure displacement is a common approach for analysing motion, loading, and load distribution in biomechanical research. Unlike gait analysis, the progression of force during boxing punches represents a relatively new area of investigation. Understanding centre of pressure displacement and the distribution of forces across the fist during a punch is essential for examining both the effects of impact on the hand’s biological structures and the technical biomechanical factors underpinning punching performance [12]. Punch force was assessed using the Hoteam Boxing Force Sensor Target Machine [13], which outputs impact values in kilograms. These values were converted to Newtons. While not equivalent in accuracy to laboratory-grade motion capture or force-measuring targets, the device offered consistent output during pilot testing and was deemed suitable for applied boxing assessments. Punch velocity was not measured as the focus of this study focused on kinetic-rather than kinematic-variables.

### 2.5. Fatigue Protocol

To simulate the lower limb fatigue experienced during boxing competition, participants completed two rounds of a structured bodyweight circuit. Participants performed the exercises at a self-selected pace. Each round lasted 4.5 min and included the following exercises:

Static Wall Sit (30 s).

Standing Calf Raises (30 s).

Bodyweight Squats (30 s).

Box Step-Ups (30 s).

Walking Lunges (30 s).

Each exercise was followed by a 30 s static wall sit. A 30 s active recovery period separated the two rounds, resulting in a total circuit duration of 9 min and 30 s. Participants continued the protocol until they either reached a rating of perceived exertion (RPE) of ≥18 on the Borg 6–20 scale or were physically unable to continue. Exercise selection was based on previous combat sports fatigue models [14].

Before and after the fatigue protocol, participants performed two sets of three maximal-effort punches for each of the following types: jab, cross, hook and uppercut. Technique was maintained across efforts, and each punch was executed with maximal intent.

### 2.6. Punch Selection and Categorisation

For each punch type, the highest and lowest force efforts were identified for each participant to examine intra-individual variability. These values were extracted from six repetitions (three per pre/post condition) per punch type. The strongest efforts were retained for further GRF and RFD analysis, while the weakest efforts were used to compare punch output changes under fatigue.

### 2.7. Data Processing and Statistical Analysis

GRF and RFD values were computed across each of the four defined punch phases. Punch force values were manually recorded from the Hoteam sensor and averaged across the three repetitions per punch type. Descriptive statistics (Mean ± SD) were calculated for all dependent variables. Strongest and weakest punch forces (Mean ± SD) were also reported.

Paired-sample t-tests were used to assess pre- vs. post-fatigue differences in GRF, RFD and punch output. The difference between the two means was examined. Cohen’s D (d) was used to express the size of the effect of the pre- vs. post-fatigue differences with effect size thresholds set as 0.2 = small, 0.5 = medium and 0.8 = large [15]. Additional paired-sample t-tests were conducted to compare fatigue-related changes between the strongest and weakest punches. Statistical significance was set at *p* < 0.05.

## 3. Results

### 3.1. Ground Reaction Forces Across All Punches (Pre-Fatigue)

Rear and front leg vertical ground reaction forces (GRFs) were recorded across movement phases during the non-fatigued condition. Rear leg GRF consistently peaked during the drive phase and declined sharply by impact across all punch types. For the jab, rear GRF reached 1243.41 ± 494.64 N during drive and dropped to 44.53 ± 33.07 N at impact. The front leg GRF increased from 362.44 ± 59.78 N at base to 418.47 ± 383.74 N at impact.

In the cross, rear leg GRF peaked at 1135.53 ± 363.79 N during drive and reduced to 90.88 ± 52.70 N at impact. The front leg GRF ranged from 442.22 ± 119.88 N at base to 472.97 ± 280.90 N at impact. For the left and right hooks, peak rear drive GRFs were 1040.68 ± 389.90 N and 1103.82 ± 478.79 N, with impact values of 97.49 ± 52.41 N and 83.37 ± 36.32 N, respectively. Corresponding front impact GRFs were 453.68 ± 288.70 N and 432.17 ± 266.22 N.

Rear drive GRF for the left and right uppercuts was 963.83 ± 312.14 N and 1028.22 ± 337.17 N, respectively, dropping to 64.32 ± 41.79 N and 85.15 ± 47.62 N at impact. Front leg GRF at impact was 408.24 ± 197.34 N (left uppercut) and 425.09 ± 230.45 N (right uppercut).

### 3.2. Punch Output Across All Punches (Pre-Fatigue)

Pre-fatigue punch force output varied across punch types. The cross produced the highest mean force (1475.42 ± 261.40 N), followed by the left hook (1374.38 ± 203.13 N), right hook (1326.31 ± 188.12 N), right uppercut (1241.95 ± 167.52 N), jab (1126.25 ± 291.67 N) and left uppercut (1007.49 ± 120.06 N).

Weakest punch efforts were also identified for intra-individual comparison. The weakest jab averaged 900.56 ± 218.52 N, while the weakest cross measured 1196.82 ± 278.35 N. Weak left and right hooks produced 1024.16 ± 189.73 N and 999.64 ± 186.39 N, respectively. The lowest forces were seen in the left uppercut (736.73 ± 143.69 N) and right uppercut (973.15 ± 180.52 N). The mean punch force across all pre-fatigue efforts was 1275.30 ± 251.43 N.

### 3.3. Rate of Force Development Across All Punches (Pre-Fatigue)

The left hook produced the highest mean RFD (2676.91 ± 1387.60 N/s), followed by the jab (2547.45 ± 1172.35 N/s) and the cross (2517.17 ± 1541.06 N/s). The right hook measured 2326.94 ± 1279.71 N/s. RFD was lowest for the left uppercut (1781.53 ± 1185.00 N/s) and right uppercut (1561.46 ± 953.19 N/s). Straight punches and hooks exhibited greater explosive force characteristics than uppercuts.

### 3.4. Ground Reaction Forces Across All Punches (Post-Fatigue)

Post-fatigue GRF patterns followed similar trends, with notable reductions at impact. For the jab, rear GRF during drive was 1370.62 ± 403.26 N and dropped to 578.69 ± 349.74 N at impact. Front leg GRF ranged from 374.81 ± 104.83 N at base to 338.55 ± 316.66 N at impact.

In the cross, rear drive GRF increased to 1324.59 ± 371.12 N, and rear impact dropped to 644.93 ± 301.28 N. Front leg values ranged from 345.17 ± 95.30 N to 500.68 ± 305.33 N. The left and right hooks recorded rear drive GRFs of 1125.18 ± 374.77 N and 1173.55 ± 449.99 N, with rear impact at 596.24 ± 283.10 N and 548.62 ± 288.70 N, respectively. Front leg impact values were 461.47 ± 278.20 N (left hook) and 455.61 ± 267.53 N (right hook).

The left and right uppercuts produced rear drive GRFs of 1061.29 ± 312.88 N and 1059.45 ± 348.36 N, with rear impact values of 499.90 N and 498.53 N. Front impact GRFs were 428.08 ± 198.47 N (left) and 443.91 ± 225.64 N (right).

### 3.5. Punch Output Across All Punches (Post-Fatigue)

Punch force declined across all types as the effect size ranged from extremely small (i.e., very close to zero/practically negligible) to medium. The jab decreased to 1133.05 ± 238.56 N (–0.6%, *p* = 0.111, d = –0.026), the cross to 1369.48 ± 190.01 N (–7.18%, *p* = 0.029, d = 0.464), and the left hook to 1261.57 ± 202.80 N (–8.21%, *p* = 0.0049, d = 0.556). The right hook declined to 1295.90 ± 166.64 N (–2.29%, *p* = 0.126, d = 0.171). The left uppercut fell to 968.25 ± 125.80 N (–3.89%, *p* = 0.292, d = 0.319), and the right uppercut to 1174.26 ± 164.42 N (–5.45%, *p* = 0.013, d = 0.408).

In the weakest punch group, jab output fell to 811.29 ± 353.49 N (–9.91%, *p* = 0.326, d = 0.304), cross to 1039.86 ± 220.77 N (–13.11%, p = 0.067, d = 0.625), left hook to 938.82 ± 126.31 N (–8.33%, *p* = 0.083, d = 0.530), and right hook to 956.48 ± 135.08 N (–4.32%, *p* = 0.192, d = 0.265). The left uppercut dropped to 634.71 ± 126.82 N (–13.85%, *p* = 0.056, d = 0.753), and the right uppercut to 876.03 ± 134.67 N (–9.98%, *p* = 0.016, d = 0.610). The difference between the pre and post groups ranged from small to large effects.

### 3.6. Rate of Force Development Across All Punches (Post-Fatigue)

Post-fatigue RFD showed variable responses. The jab increased to 3420.59 ± 1178.30 N/s (+34.28%, *p* = 0.0116). The cross showed a significant decline to 2029.56 ± 1594.39 N/s (–24.03%, *p* = 0.027). The left and right hooks declined to 2327.65 ± 2339.72 N/s (–13.05%, *p* = 0.664) and 2158.99 ± 1798.33 N/s (–7.22%, *p* = 0.620), respectively. The left uppercut increased to 2307.95 ± 1425.87 N/s (+29.55%, *p* = 0.081), while the right uppercut showed the largest increase, rising to 2324.30 ± 1372.39 N/s (+48.85%, *p* = 0.021).

### 3.7. Comparative Effects of Fatigue on Strongest and Weakest Punches

**Figure 2:**
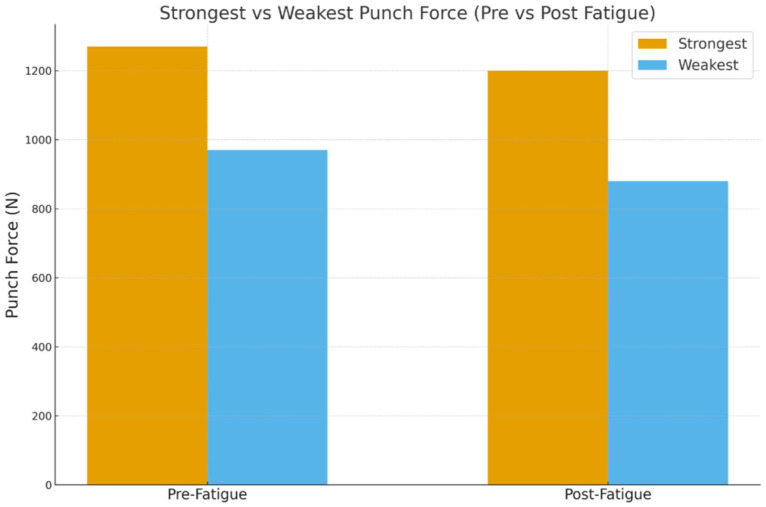
Histogram representation of the Comparative Effects of Fatigue on the Strongest and Weakest Punches.

The strongest punches declined from 1258.63 ± 170.79 N to 1200.42 ± 141.82 N (–4.63%, *p* = 0.027). The weakest punches showed a greater drop, from 971.84 ± 151.32 N to 876.20 ± 141.20 N (–9.84%, *p* = 0.001) (Figure 2). Among the weakest punches, the left uppercut (–13.85%) and cross (–13.11%) exhibited the largest reductions.

### 3.8. Calibration and Validation of the Hoteam Boxing Force Sensor

The Hoteam Boxing Force Sensor was calibrated and validated prior to data collection. Static calibration was performed using traceable weights (0 kg–150 kg) and a Kistler Force Plate to generate a calibration curve across the full measurement range; the calibration was linear across the operational range (R^2^ = 0.98). Dynamic validation consisted of synchronous recordings of 10 controlled (20 kg) strikes using a mechanical striker performed by a laboratory technician onto a calibrated Kistler force platform. The results produced a coefficient of variation of 4.57%.

## 4. Discussion

This study investigated the role of lower limb kinetics, specifically ground reaction force (GRF) and rate of force development (RFD), in boxing punch performance and examined how these variables respond to fatigue. Six punch types were assessed using force plates and a punch force sensor in both pre and post fatigue conditions. The results confirmed that GRF peaked during the drive phase, particularly through the rear leg and that the cross and left hook produced the highest punch outputs. Following fatigue, both punch force and GRF generally declined, although RFD responses varied [7,9].

A notable strength of this study was the intra-individual comparison between each participant’s strongest and weakest punches across all six strike types. These comparisons revealed significant variability in performance, with the weakest punches consistently producing markedly lower outputs. For example, the left hook exhibited the greatest decline post-fatigue (−8.21%) and the largest difference between strongest and weakest trials (−22.8%). These findings reinforce the importance of efficient kinetic chain sequencing and suggest that this efficiency is vulnerable to disruption under fatigue [6,16], supporting earlier research on rear-leg contributions and whole-body coordination in effective striking [9].

### 4.1. Lower Limb Kinetics and Punch Performance

Peak GRF consistently occurred during the drive phase, with the rear leg contributing most significantly to vertical (Fz) and anterior-posterior (Fy) force production. Pre-fatigue, the cross delivered the highest punch force (1475.42 N), GRF (947.54 N), and RFD (3973.38 N/s), followed closely by the left hook (1374.38 N, 868.53 N, and 3797.01 N/s, respectively). These findings align with Stanley et al. [7] and Liu et al. [10], who reported elevated GRF values in experienced boxers during rotational punches.

The jab recorded the lowest punch force (1126.25 N) and GRF (791.76 N) but produced high RFD (2919.91 N/s), indicating a compensatory reliance on rapid force production rather than total force. This supports McGill et al. [3], who highlighted the importance of upper-body neuromuscular coordination in short-range strikes.

Uppercuts, particularly the left uppercut, yielded the lowest GRF (700.28 N) and RFD (2559.28 N/s), potentially due to their more vertical trajectory and limited leg drive involvement [6]. These variations in kinetic output highlight the distinct mechanical demands of each punch type and advocate for punch-specific training approaches [10,17,18].

### 4.2. Fatigue Effects on Kinetics and Output

Post-fatigue analysis revealed reductions in GRF across all punches, with the greatest losses observed in the left hook (−68.86 N) and right uppercut (−61.17 N). Punch output also declined, particularly in the left hook (−8.21%), cross (−7.18%) and right hook (−6.25%), with an overall output reduction of 4.26% (*p* = 0.027). These results are consistent with prior findings that fatigue diminishes ground force production and disrupts kinetic chain transfer [19,20].

Interestingly, RFD did not uniformly decrease. The jab and right uppercut displayed increased RFD values post-fatigue (+255.67 N/s and +135.43 N/s, respectively), possibly reflecting a neuromuscular strategy to maintain punch velocity through faster muscle activation. This is in line with the force-velocity relationship, where diminished force capacity can be offset by increased contraction speed [18,21,22]. While potentially beneficial for preserving speed, these adjustments may compromise mechanical efficiency or elevate injury risk [23].

Differences in fatigue responses among punches indicate varied dependence on the lower limbs. These distinctions should guide fatigue-specific conditioning, particularly when structuring high-intensity rounds or sparring protocols [24,25]. Conditioning aimed at the cross and hooks may require enhanced focus on sustaining lower limb force output under fatigue.

### 4.3. Strong Versus Weak Punch Comparisons

This study’s intra-individual comparison of the strongest versus the weakest punches offers unique insights. Across participants, weaker punches consistently underperformed. The left hook demonstrated a 22.8% performance gap with notable differences also seen in the left uppercut (−13.85%), right uppercut (−9.98%), jab (−9.31%) and right hook (−9.16%). These results emphasise that improving consistency across punch types may be as crucial as raising peak performance [25,26].

Although GRF and RFD data were only collected for the strongest punches, punch output data alone revealed meaningful discrepancies. These may stem from subtle differences in technique, neuromuscular readiness, or psychological focus. Steib et al. [23] noted that fatigue-related postural instability could affect the consistency of force application, reinforcing this interpretation.

These findings have practical coaching implications. Monitoring weak punch output-not just peak performance-could enhance training specificity, reduce variability and improve overall striking reliability [27].

### 4.4. Methodological Strengths and Limitations

This study employed high-frequency (1000 Hz) force plates and punch-specific force sensors to provide detailed kinetic analysis. Dividing punches into four phases, Base, Load, Drive and Impact, enabled precise segmentation of movement. The fatigue protocol was designed to replicate lower limb exertion typical of a boxing round, and the use of the Borg scale (RPE ≥ 18) ensured sufficient fatigue was reached.

The study did not incorporate electromyographic (EMG) measures, which limits the interpretation of underlying neuromuscular activation. However, the primary focus was on kinetic force-related variables, which were reliably captured using the available equipment. While the Hoteam device lacks formal lab calibration, it provided consistent relative output supporting its use for comparative force assessments in applied sport environments. This methodological trade-off reflects both the practical constraints and applied nature of the research setting. Other limitations were the small sample size (n = 10), which limits generalizability and statistical power. The small sample size (n = 10) inevitably reduces the statistical power of the study, increasing the likelihood of Type II error and limiting the ability to detect subtle or moderate effects. As a result, statistically non-significant findings should be interpreted cautiously, as they may reflect insufficient power rather than the absence of a true effect. Additionally, the limited number of participants restricts the generalizability of the results. With such a small cohort, individual variability may disproportionately influence group outcomes, and the findings may not fully represent the broader population of athletes or individuals with differing characteristics. While the study offers valuable preliminary insights, future research with larger, more diverse samples is necessary to confirm these patterns and strengthen external validity. The RFD results showed very high variability (i.e., large standard deviation). This may have been attributed to differences in technique among the boxers and/or the dynamic nature of the parameter.

Additionally, extensive post-processing was required: 960 punch phases were identified and segmented into 4800 subframes for averaging, requiring approximately 70 h of manual labour. While time-intensive, this enhanced data accuracy reinforced the robustness of the analysis. Future studies should expand sample size, include professional athletes and integrate motion capture and EMG data. This could improve understanding of trunk and lower limb coordination during punch execution under fatigue.

The Hoteam Boxing Force Sensor is a practical, wearable field device that enables ecologically valid testing of boxing punches. However, compared with laboratory-grade force plates and load cells, it has lower absolute accuracy and a narrower effective bandwidth. While we calibrated the sensor across the expected force range and demonstrated strong within-participant repeatability, direct comparisons with laboratory references yielded a small systematic bias and wider limits of agreement. Consequently, the Hoteam sensor is most appropriate for detecting relative changes (for example, pre- vs. post-fatigue) and between-condition comparisons rather than for obtaining gold-standard absolute force values. We minimized limitations through careful calibration and standardised sensor placement; however, consistency of gloves and hand wrapping across all participants was not controlled for. Participants were given the flexibility to use their own boxing gloves and hand wraps.

It must be acknowledged that using a vertical force plate integrated with the Vicon Motion System would have been preferred. However, a force plate, which is considered the gold standard, has inherent limitations [28,29]. Its measurement error is not zero. Research has shown that errors increase particularly when the normal forces applied perpendicular to the plate’s surface are small relative to the horizontal forces and/or when the point of force application is positioned near the edge of the plate.

### 4.5. Practical Applications and Future Research

The findings offer clear applications for boxing coaches and sports scientists. Crosses and hooks rely heavily on rear leg drive and should be targeted with strength and power training interventions, such as Olympic lifts, plyometrics, jump squats and resisted shadowboxing [6,18]. Fatigue resistance should also be developed through HIIT, circuit training and fatigued bag work [30,31,32,33].

Further practical implications include that the observed reductions in punch force and alterations in lower-limb kinetics following fatigue emphasize the need for training strategies that enhance the endurance capacity of the kinetic chain. Incorporating targeted lower-limb conditioning, technical drills performed under controlled fatigue, and systematic monitoring of force output may help preserve striking efficiency during high-intensity bouts. These findings underscore the importance of fatigue-informed training load management to optimize performance and minimize the risk of technique breakdown in competitive situations.

Identifying intra-individual performance gaps can guide technique refinement and reduce variability [26]. Future research should assess whether interventions like velocity-based or isometric training can attenuate fatigue-induced performance loss. Longitudinal studies (including predictive simulation studies) exploring adaptations to periodised programs aimed at improving rear leg output may provide further insights [34]. Motion capture and EMG should also be employed to deepen understanding of neuromuscular adaptations and kinetic chain sequencing-particularly for complex strikes like hooks and uppercuts [16].

## 5. Conclusions

This study examined the importance of lower limb kinetics in boxing punch performance and assessed the effects of fatigue on GRF, RFD and punch force across six punch types. Findings confirmed that rear leg drive is critical to producing high punch output, particularly in rotational strikes such as the cross and hook. Fatigue significantly reduced punch force and GRF, while RFD showed variable responses, with some punches demonstrating compensatory increases. Intra-individual comparisons revealed that weaker punches experienced greater performance declines, emphasising the importance of training for consistency as well as peak output. These insights highlight the value of targeted lower limb conditioning and fatigue-specific strategies to preserve kinetic chain efficiency. Future research should incorporate larger samples, EMG and motion capture to enhance understanding of neuromuscular coordination during fatigued striking.

## Data Availability

The data that support the findings of this study are not openly available due to reasons of sensitivity and are available from the corresponding author upon reasonable request. Data are located in controlled access data storage at the University of the West of Scotland.

## References

[1] Da Broi M., Al Awadhi A., Voruz P., Nouri A., Schaller K. (2023). The spectrum of acute and chronic consequences of neurotrauma in professional and amateur boxing: A call to action is advocated to better understand and prevent this phenomenon. Brain Spine.

[2] Donnelly R.R., Ugbolue U.C., Gao Y., Gu Y., Dutheil F., Baker J.S. (2023). A systematic review and meta-analysis investigating head trauma in boxing. Clin. J. Sport Med..

[3] McGill S.M., Chaimberg J.D., Frost D.M., Fenwick C.M.J. (2010). Evidence of a double peak in muscle activation to enhance strike speed and force: An example with elite mixed martial arts fighters. J. Strength Cond. Res..

[4] Whiting W.C., Gregor R.J., Finerman G.A. (1988). Kinematic analysis of human upper extremity movements in boxing. Am. J. Sports Med..

[5] Zazryn T., Cameron P., McCroy P. (2006). A prospective cohort study of injury in amateur and professional boxing. Br. J. Sports Med..

[6] Dunn E.C., Humberstone C.E., Franchini E., Iredale K.F. (2022). The effect of fatiguing lower-body exercise on punch forces in highly trained boxers. Eur. J. Sport Sci..

[7] Stanley E., Thomson E., Smith G., Lamb K.L. (2018). An analysis of the three-dimensional kinetics and kinematics of maximal effort punches among amateur boxers. Int. J. Perform. Anal. Sport.

[8] Lenetsky S., Brughelli M., Nates R.J., Neville J.G., Cross M.R., Lormier A.V. (2020). Defining the phases of boxing punches: A mixed-method approach. J. Strength Cond. Res..

[9] Dinu D., Louis J. (2020). Biomechanical analysis of the cross, hook, and uppercut in junior vs. elite boxers: Implications for training and talent identification. Front. Sports Act. Living.

[10] Liu Y., Wang Y., Zhang J., Zhang Y. (2022). Biomechanics of the lead straight punch of different level boxers. Front. Physiol..

[11] Perlinski J., Sikorski W., Wochna K., Raczek N. (2024). Gait analysis of male professional boxers. Balt. J. Health Phys. Act..

[12] Menzel T., Potthast W. (2021). Validation of a novel boxing monitoring system to detect and analyse the centre of pressure movement on the boxer’s fist. Sensors.

[13] Boxing Strength Tester-Punch Force Sensor|Equipment Wall Mounted Boxing Boxer | Tester Boxing Training Sandbag Vent Target|Wall Punch Pad Boxing Punching Bag Machine for Adult Kid: Amazon.co.uk: Sports & Outdoors. https://www.amazon.co.uk/Boxing-Strength-Tester-Equipment-Training/dp/B0FJPS79Y2/ref=sr_1_2?crid=33AQUHWQJXOEJ&dib=eyJ2IjoiMSJ9.xr1DY1Qpz9O4fZc0l2laFUVZ6VR8SDLaa3SlrWnS7fRrtQT80iA8V-bWRrnTYVkpoFiTCXqm8q3dMXB6RoTxAwT0wrhWVPZBOUWtz1Gi4ZwqVPbdaFH-AwtfzcqN5uiDtC4x8ryk7WNYQgL-AdlnmHwDjBWqsS5LVj7Nn6gjP520xFXygpaweh56r48Bbd9UIg-8A3gvNYe8O_lV3wYkqd0q6kM5t4Kg7mj8n79Abwb4hkV3T7ekLxuZztdbaTmH8VAAOgyHAib72sQ3ZzakZW4aqS0kWuwbkqklljb0Ep8.QFzOS3vF-eQhDuW2wHxNoLB4Kk0hMipqWJO-Qj_6LHE&dib_tag=se&keywords=boxing+punch+tester&qid=1764391337&s=sports&sprefix=boxing+punch+tester%2Csports%2C210&sr=1-2.

[14] Sayyadi P., Razeghi M., Jalali M. (2024). The effectiveness of fatigue on repositioning sense of lower extremities: Systematic review and meta-analysis. BMC Sports Sci. Med. Rehabil..

[15] Cohen J. (2013). Statistical Power Analysis for the Behavioral Sciences.

[16] Almansoof B., Batal M., Youm Y. (2021). Development of a musculoskeletal model for lower limb motion analysis during boxing punches. Procedia Comput. Sci..

[17] Yi W., Chen C., Zhou Z., Cui W., Wang D. (2022). Acute effects of ballistic versus heavy-resistance exercises on countermovement jump and rear-hand straight punch performance in amateur boxers. BMC Sports Sci. Med. Rehabil..

[18] Loturco I., Artioli G.G., Kobal R., Gil S., Franchini E., Nakamura F.Y. (2016). Strength and power qualities are highly associated with punching impact in elite amateur boxers. J. Strength Cond. Res..

[19] Tong-Iam R., Rachanavy P., Lawsirirat C. (2017). Kinematic and kinetic analysis of throwing a straight punch: The role of trunk rotation in delivering a powerful straight punch. J. Phys. Educ. Sport.

[20] Haralabidis N., Saxby D.J., Pizzolato C., Needham L., Cazzola D., Minahan C. (2020). Fusing Accelerometry with Videography to Monitor the Effect of Fatigue on Punching Performance in Elite Boxers. Sensors.

[21] Cormie P., McGuigan M.R., Newton R.U. (2011). Developing maximal neuromuscular power: Part 1—Biological basis of maximal power production. Sports Med..

[22] Cormie P., McGuigan M.R., Newton R.U. (2011). Developing maximal neuromuscular power: Part 2—Training considerations for improving maximal power production. Sports Med..

[23] Steib S., Hentschke C., Welsch G., Pfeifer K., Zech A. (2013). Effects of fatiguing treadmill running on sensorimotor control in athletes with and without functional ankle instability. Clin. Biomech..

[24] Sánchez-Ramírez C., Cid-Calfucura I., Hernandez-Martinez J., Cancino-López J., Aedo-Muñoz E., Valdés-Badilla P., Franchini E., García-García J.M., Calvo-Rico B., Abián-Vicén J. (2025). Submaximal Accentuated Eccentric Jump Training Improves Punching Performance and Countermovement Jump Force–Time Variables in Amateur Boxers. Appl. Sci..

[25] Dunn E.C., Humberstone C.E., Franchini E., Iredale K.F., Blazevich A.J. (2022). Relationships Between Punch Impact Force and Upper- and Lower-Body Muscular Strength and Power in Highly Trained Amateur Boxers. J. Strength Cond. Res..

[26] Mosler D., Kacprzak J., Wąsik J. (2024). The influence of effective mass on the striking force of lead jab and rear cross punches of boxers. Appl. Sci..

[27] Lenetsky S., Brughelli M., Nates R.J., Cross M.R., Lormier A.V. (2018). Variability and Reliability of Punching Impact Kinetics in Untrained Participants and Experienced Boxers. J. Strength Cond. Res..

[28] Schmiedmayer H.B., Kastner J. (1999). Parameters influencing the accuracy of the point of force application determined with piezoelectric force plates. J. Biomech..

[29] Chesnin K.J., Selby-Silverstein L., Besser M.P. (2000). Comparison of an in-shoe pressure measurement device to a force plate: Concurrrent validity of center of pressure measurements. Gait Posture.

[30] Herrera-Valenzuela T., Carter J., Leiva E., Valdés-Badilla P., Ojeda-Aravena A., Franchini E. (2021). Effect of a short HIIT program with specific techniques on physical condition and activity during simulated combat in national-level boxers. Sustainability.

[31] Usher A., Babraj J. (2025). Impact of sprint interval training on post-fatigue mitochondrial rate in professional boxers. Eur. J. Appl. Physiol..

[32] Nukeaw A., Boonrod W. (2020). Effect of Supplemented Muay Thai Circuit Training Program on Maximal Oxigen Uptake and Physical Performance in Professional Muay Thai Boxers. J. Sports Sci. Health.

[33] Niu Z., Huang Z., Zhao G., Chen C. (2024). Impact of three weeks of integrative neuromuscular training on the athletic performance of elite female boxers. PeerJ.

[34] Haralabidis N., Colyer S.L., Serrancolí G., Salo A.I.T., Cazzola D. (2022). Modifications to the net knee moments lead to the greatest improvements in accelerative sprinting performance: A predictive simulation study. Sci. Rep..

